# Exploring the Role of Community Pharmacists in Pain Management: Enablers and Challenges

**DOI:** 10.3390/pharmacy12040111

**Published:** 2024-07-18

**Authors:** Syed Hassan Mujtaba, Parisa Gazerani

**Affiliations:** 1 Department of Life Sciences and Health, Faculty of Health Sciences, Oslo Metropolitan University, 0130 Oslo, Norway; 2 Department of Health Science and Technology, Faculty of Medicine, Aalborg University, 9260 Gistrup, Denmark

**Keywords:** pharmacists, community pharmacist, pharmacy, pain, pain management, rational pharmacotherapy, non-pharmacological strategies, multidisciplinary, primary healthcare

## Abstract

Pain is a common complaint, and the consumption of analgesics is prevalent. Community pharmacists, as primary contact points for patients, can play a crucial role in guiding patients toward rational pharmacotherapy or alternative pain management strategies. However, there are no specific educational curricula or standard guidelines to support this role, and the perception of this potential role is not well known. We conducted an anonymous online questionnaire among community pharmacists in Norway to assess their knowledge, perspectives, and willingness to engage in pain care. The survey also explored potential facilitators and barriers, and the use of any current guidelines. Seventy-one community pharmacists participated from various regions in Norway. Findings revealed that community pharmacists felt knowledgeable and willing to engage in pain management but anticipated barriers such as time constraints and a lack of standard guidelines. Participants also highlighted the need for better collaboration with other healthcare professionals and continuous professional development to enhance their role. To optimize the role of community pharmacists in pain management, therefore, integrating them into multidisciplinary healthcare teams, minimizing barriers, and providing continuous education and standard guidelines seem essential. This approach can empower community pharmacists and improve pain management outcomes.

## 1. Introduction

### 1.1. Pain and Pain Management

Pain [1] is a complex phenomenon influenced by biological, psychological, emotional, and cultural factors, making it highly individualized [2]. Chronic pain represents a substantial global health challenge, affecting one in five adults [3]. Surveys in Europe reveal that 25% to 35% of adults report chronic pain [4]. In Norway, the prevalence is around 30%, leading to significant economic burdens due to prolonged sick leave and disability [5,6]. Chronic pain impacts physical and emotional well-being and exerts financial strain on healthcare systems and the broader societal economy.

Chronic pain affects all demographic groups but disproportionately impacts older adults, especially those over 65, causing distress, social isolation, and financial costs [7,8,9]. Women report more intense pain and greater disability than men [10,11]. Chronic pain often coexists with mental health issues, necessitating a comprehensive, patient-centered approach that addresses compliance, personalized treatment, psychological well-being, and rational medication use [12].

Effective pain management requires well-defined protocols, adequate knowledge among healthcare providers, teamwork, managing adverse effects, patient education, and guidelines for the rational use of analgesics and alternative pain relief strategies [13]. However, a comprehensive approach is often lacking, highlighting the need for improvements in interprofessional communication and patient education. A multimodal strategy integrating pharmacological and non-pharmacological treatments is essential [14]. This approach recognizes the multifaceted nature of chronic pain and emphasizes employing a combination of treatment modalities, including a multidisciplinary team where the role of pharmacists, among other healthcare professionals, must be recognized [15,16,17,18].

### 1.2. Role of Pharmacists in Pain Management

Pain management through a multidisciplinary approach incorporates the expertise of diverse healthcare professionals to evaluate a patient’s condition and develop an inclusive care plan. The team generally includes primary care physicians, anesthesiologists, psychologists, nurses, physical and occupational therapists, surgeons, neurologists, and pharmacists [19]. This collaborative effort ensures a holistic pain management approach, addressing both physical and psychological facets effectively. In Norway, patients with severe chronic pain are entitled to prioritized healthcare services within multidisciplinary pain clinics [4,20].

Despite the emphasis on proper pain management in hospitals, outpatient studies are limited, and community pharmacists’ roles are underexplored [21]. Community pharmacists often interact more frequently with patients than primary care providers, presenting a special opportunity to monitor patients, provide medication counseling, address chronic pain queries, and share information on non-pharmacological pain relief strategies. Their consistent presence in patients’ healthcare routines enables a more thorough and individualized approach to chronic pain management. Given the complexity of chronic pain management and the prevalence of comorbidities, individuals often require complex drug regimens. Pharmacists, as readily accessible medication experts, play a vital role in helping patients manage their treatment plans, considering their chronic pain, coexisting health issues, concurrent medications, and individual preferences, expectations, and goals [18].

Engaging specialized clinical pharmacists in pain management has numerous advantages, including alleviating physicians’ workloads, optimizing opioid use, reducing medication-related adverse effects, and decreasing pain intensity [22]. Collaborative approaches between pharmacists and patients are associated with enhanced pain management outcomes, highlighting pharmacists’ significant role in improving patient care and well-being [23]. Integration of clinical pharmacists in pain management leads to decreased medication dosages, enhanced adherence to best practices, optimization of medication therapy, and improved patient access and safety [24]. Pharmacist participation in inpatient pain management consultation services yields favorable outcomes, including positive impacts on pain scores and functional improvements among patients [25]. Studies highlight the role of clinical pharmacists in identifying and addressing medication-related problems in chronic pain management, contributing to enhanced patient care and safety [26].

While the role of clinical pharmacists in pain management has received significant attention, the role of community pharmacists remains underdeveloped. Community pharmacists play a pivotal role in pain care, with individuals predominantly utilizing their services for pain-related issues [17,18]. They contribute significantly by providing guidance, recommending suitable over-the-counter (OTC) pain relief options, both pharmacological and non-pharmacological, and facilitating referrals when necessary, enhancing the overall quality of pain management services. Additionally, community pharmacists play a critical role in recognizing and mitigating drug interactions, particularly important in chronic pain management where polypharmacy is prevalent [27]. Despite these contributions, research examining the knowledge and attitudes of community pharmacists regarding pain management is limited.

A 2015 scoping review [28] emphasizes the importance of community pharmacists in managing pain in adults. This study outlines how pharmacists’ involvement in pain management can lead to better patient outcomes through their expertise in medication management and patient education [28]. Another study from the same year [29] presents barriers and facilitators affecting pharmacists’ roles in pain management, finding that while pharmacists have significant potential, they often face barriers such as lack of time, insufficient training, and limited collaboration with other healthcare professionals [29]. A 2018 study [30] discusses the evolving role of pharmacists in pain management, highlighting their responsibilities in monitoring medication use, preventing adverse drug events, and providing patient education on pain management strategies. This article shows pharmacists’ role in managing complex pain cases involving multiple medications and comorbidities, significantly enhancing patient care and safe analgesic use [30]. It suggests that the role of community pharmacists in pain management can be clarified through structured training programs that promote enhanced communication between pharmacists and other healthcare professionals. These studies collectively demonstrate that community pharmacists can be integral to effective pain management, contributing through medication management, patient education, and collaboration with other healthcare professionals. By addressing identified barriers, their role can be further optimized to improve patient outcomes in pain management.

We could not find any studies specifically addressing this topic in Norway. Consequently, we aimed to fill this gap by exploring the current knowledge, attitudes, and practices of community pharmacists in Norway regarding pain management and identifying barriers and facilitators to enhance their role in pain care. We hypothesize that community pharmacists possess the necessary knowledge and skills to significantly contribute to pain management and improve patient outcomes. We focused on educational efforts, personalized pain management, and collaboration with healthcare professionals. Our goal was to explore how community pharmacists perceive their contribution to pain management, gauge their confidence in these roles, and pinpoint areas of improvement. By assessing their current practices along with perceived obstacles and facilitators, we aimed to create an evidence-based foundation for strategies to reinforce the role of community pharmacists as integral team members in pain management and advocates for patient well-being.

## 2. Materials and Methods

### 2.1. Study Design 

A quantitative, cross-sectional study was conducted to investigate the knowledge, perspectives, and self-reported competencies of Norwegian community pharmacists in pain management. Data collection was carried out using an online, anonymous questionnaire [31], targeting community pharmacists in Norway for fast and efficient data collection. To maintain anonymity and ensure data confidentiality, no personal or sensitive information was collected. The questionnaire was designed in Norwegian, the legal language of Norway, and participants provided voluntary consent to share their anonymous responses. Utilizing the “Nettskjema” platform facilitated easy participation, with respondents accessing the questionnaire through a shared link. On average, completion of the questionnaire took approximately 12 min.

### 2.2. Study Population

In line with the specific focus of this study, the targeted population was defined as pharmacists working in either chain or private community pharmacies in Norway, with the additional requirement of being a registered pharmacist in Norway. These inclusion criteria defined the eligible participants. This specific demographic focus was also clearly stated in the questionnaire’s description to ensure the relevance and precision of data collection. 

### 2.3. Construction of the Questionnaire

Given the lack of a pre-existing standardized questionnaire, a new one was crafted for this study. Drawing insights from the existing literature, a systematic review was conducted to ensure the relevance and currency of the questions, also identifying gaps in the field. This process guided the questionnaire’s development starting with a brief introduction outlining the study’s purpose, emphasizing anonymity, voluntary participation, and consent for data use. Participants were thanked for their engagement upon completion. Structurally, it comprised four sections tailored to gather specific insights from community pharmacists. Combining open-ended and closed-ended questions facilitated comprehensive data collection [32].

Section 1: Sociodemographic Data

This section of the questionnaire served to establish a demographic profile of the participants, covering essential aspects such as gender, age, work experience, education level, and geographic location within Norway. Comprising seven closed-ended questions, it began by prompting respondents to indicate their gender, followed by categorizing them into age groups. Work experience as a pharmacist was then assessed, with options ranging from newly graduated to over 10 years of experience. Education level was queried next, followed by work location within Norway, covering various regions. Importantly, participants were asked about their training in pain management, with options to specify the source if applicable. The section concluded by inquiring about the origin of their pharmacy degree, whether obtained in Norway or abroad, with the latter requiring specification of the country.

Section 2: Knowledge, Skills, and Competencies

Section two of the questionnaire aimed to evaluate the knowledge, skills, and competencies of community pharmacists concerning pain management and various types of analgesics and their use. Comprising ten questions, it focused on both non-opioid and opioid analgesics. For non-opioids, mainly OTC, simple analgesics, such as non-steroidal anti-inflammatory drugs (NSAIDs), co-analgesics such as adjuvants (for example, antidepressants), and a combination of analgesics were considered. Responses were recorded on a 5-point Likert scale, ranging from “Strongly Disagree” to “Strongly Agree”, facilitating a nuanced assessment of pharmacists’ perspectives. All questions were closed-ended, enabling a structured evaluation of respondents’ knowledge. Topics covered included pain management protocols for children, perception of pain in infants, medication selection, use of Cognitive Behavioral Therapy (CBT) for chronic pain [33,34], analgesic effectiveness, risks of NSAIDs, the role of antidepressants in pain management, and combination analgesic therapy for improved pain control. CBT was queried due to its widespread recognition as a psychological treatment approach for chronic pain [35]. It focuses on assisting individuals in identifying negative thought patterns and substituting them with healthier, more constructive ones. In pain management, CBT techniques are frequently utilized to aid patients in developing coping strategies, stress management, and enhancing overall quality of life. Community pharmacists potentially play a crucial role in educating patients about CBT as an adjunctive therapy for chronic pain, highlighting its potential benefits alongside pharmacological interventions [36].

Section 3: Self-reported Competence

Section three of the questionnaire addressed the self-reported competence of registered community pharmacists in pain management, comprising 14 questions [32]. Most questions were closed-ended, with a few allowing for open-ended responses. The section covered various aspects, including patient counseling on OTC analgesics, non-pharmacological pain management approaches, awareness of potential drug interactions, and knowledge of guidelines and regulations related to analgesics. Questions also explored familiarity with clinical guidelines, self-assessed knowledge of controlled substances and pain management, and opinions on the need for additional training. Response formats varied: four questions utilized a 5-point Likert scale, six assessed the frequency of practices on a scale from “Low” to “Very High”, and two provided a three-option format. An additional question expanded to a four-option format, and a sub-question prompted respondents to specify reasons for their responses, ensuring a comprehensive assessment of pharmacists’ capabilities and experiences in pain management.

Section 4: Facilitators and Barriers

This section explored the factors influencing the role of community pharmacists in pain management, featuring 15 questions of mixed closed- and open-ended types. It began by addressing the unique challenges pharmacists face when dealing with chronic pain patients, including barriers like knowledge gaps, communication issues, and discomfort in advising specific demographic groups. Pharmacists could also mention other challenges not listed. The section then assessed differences observed with chronic pain patients, such as increased medication use and higher instances of anxiety or depression. Pharmacists also reflected on collaboration with other health professionals, their role in patient education, and the impact of limited pain medication sales. They were asked to rate the effectiveness of collaboration and their participation in multidisciplinary discussions. Moreover, pharmacists evaluated the impact of pharmacy workflow and staffing levels on their ability to provide comprehensive pain management advice and the importance of clear guidelines and protocols. The section concluded by soliciting pharmacists’ opinions on factors facilitating effective pain management in a community pharmacy, aiming to comprehensively understand their challenges, needs, and contributions in this domain, offering insights for improvement and development.

### 2.4. Pilot Testing of the Questionnaire

After constructing the draft questionnaire, an internal pilot test was conducted from 9 November to 13 November 2023, to gather initial feedback and ensure its effectiveness. The pilot test involved a diverse group of individuals, including pharmacists and non-pharmacists, to collect a wide range of perspectives. The main objective was to identify any necessary corrections related to question formulation, options provided, text clarity, and the time required to complete the questionnaire. Ten individuals participated in the pilot test and provided feedback, including suggestions for changes, additions, or deletions. Based on this feedback, revisions were made to certain questions and options, and some questions were removed to ensure the questionnaire could be completed within 10–12 min. These adjustments aimed to better align the questionnaire with respondents’ perspectives and understanding, enhancing its effectiveness in capturing accurate and relevant data.

### 2.5. Questionnaire Distribution and Data Collection

The final version of the questionnaire, refined based on pilot test feedback, was distributed online within the “Farmasi” social media group, dedicated to Norwegian pharmacists, with approximately 5800 members. The questionnaire remained open for seven weeks, from 29 November 2023 to 12 January 2024, allowing ample time for participation. Two reminders were sent at two-week intervals to encourage participation. Responses were automatically compiled into an Excel sheet format using the Nettskjema platform. The original questionnaire (Norwegian) and its translated version (English) are available as Supplementary Material. 

### 2.6. Ethical Considerations

Ethical considerations were upheld by ensuring anonymity and voluntariness, with no collection of personal or sensitive data. Formal ethical approval was not required from the Regional Committee for Medical and Health Research Ethics (REC) or the Norwegian Data Protection Agency due to these measures. However, the questionnaire was submitted to the Data Protection Services for Research in Norway, Norwegian Agency for Shared Services in Education and Research (SIKT) for informational purposes and feedback. Upon confirmation from SIKT (reference number 658100, 28 November 2023) regarding the absence of sensitive data collection, the questionnaire was disseminated via social media to the target audience of community pharmacists.

### 2.7. Data Handling and Statistical Analysis

After respondents completed the questionnaire, their responses were automatically compiled and organized into an Excel sheet, streamlining the data collection process and providing a comprehensive overview in a single file. For analysis, the data were imported into IBM^®^ SPSS^®^ Statistics version 29.0.1.0. Descriptive statistics were conducted to align with the research objectives. Variables were categorized based on their nature and type. Independent variables were demographic variables like gender, age, work experience, education level, work location in Norway, training in pain management, source of training, and completion of pharmacy education. Categorical variables included gender, age, work location, pain management training, and education completion location, while work experience and education level were continuous variables. Dependent variables covered respondents’ knowledge, skills, and competence in pain management, professional practices, challenges, attitudes, beliefs, and barriers and facilitators in pain management. These variables were classified as categorical.

Demographic data are tabulated with numbers and percentages, while bar and pie charts illustrate other data. We also outlined null hypotheses (Table 1) and the corresponding statistical tests, such as the chi-square test for categorical variables and ordinal regression analysis for categorical dependent and continuous independent variables. To minimize Type 1 error risk due to multiple analyses on the same dataset, a Bonferroni correction was applied to adjust the significance level, ensuring stricter criteria for determining significant associations between variables.

## 3. Results

We designed the questionnaire with the expectation of receiving at least five responses per question, aiming for a total of 235 responses. However, we ultimately received 71 responses, yielding a response rate of 30.2%. It is notable that response rates for questionnaires typically range between 30% and 50% and recent studies have indicated that the average response rate for online questionnaires stands at approximately 44.1% [37]. Despite targeting a sample size from the Facebook page of the Norwegian pharmacists, which has 5800 members, we did not achieve our expected high participant numbers. These limitations should be considered when interpreting the study results and the subsequent analysis conducted.

### 3.1. Socio-Demographic Characteristics

The details of the sociodemographic information of the 71 respondents are shown in Table 2. The questionnaire respondents were predominantly female (59.2%). In terms of age distribution, nearly half were over 32 years old (46.5%), while the 27–32 age group represented 38%, and the 21–26 age group accounted for 15.5%.

Regarding work experience, respondents varied, with the majority having less than 5 years of experience (40.8%), followed by those with over 10 years of experience (29.6%), and 5–10 years (18.3%). Recent graduates were the smallest group (11.3%).

In terms of education, most held a Bachelor’s degree in pharmacy (60.6%), followed by a Master’s degree in pharmacy (35.2%). Other educational levels were less common (4.2%), including Pharm-D and Master’s in Clinical Pharmacy.

Work locations were diverse, with the Eastern region having the highest representation (49.3%), followed by the Central region (19.7%), Northern region (12.7%), Southern region (9.9%), and Western region (8.5%).

Regarding pain management training, the majority (63.4%) had received relevant training, while 17 (23.9%) had not, and 9 (12.7%) could not recall. Primary sources of training were at the pharmacy (46%) and during university studies (44%), with other sources being less common.

Regarding pharmacy degree completion, 70.4% had completed their degree in Norway, while the remainder (29.6%) had completed their degree in countries such as Pakistan (71%), Egypt, Ethiopia, Iran, and Serbia, and two respondents did not mention their country of education.

### 3.2. Knowledge, Skills, and Competence in Pain and Pain Management 

**Figure 1:**
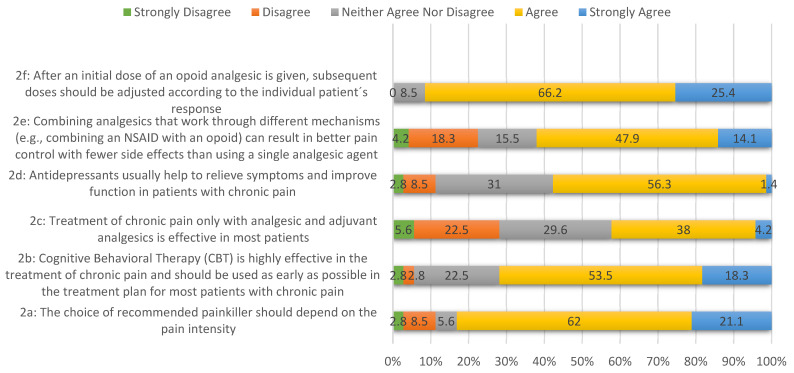
The degree of agreement and disagreement among the respondents regarding various aspects of pain management.

In Figure 1, respondents’ attitudes toward various pain management approaches are depicted. For statement 2a, 62% agree, and 21.1% strongly agree that painkiller selection should consider pain intensity, indicating a preference for tailored pain management. In statement 2b, 53.5% agree, and 18.3% strongly agree with the efficacy of Cognitive Behavioral Therapy (CBT) in treating chronic pain, supporting its early incorporation into treatment plans. Regarding chronic pain treatment with analgesics (statement 2c), opinions vary, but 38% agree that they are effective for most patients.

In statement 2d, the majority (56.3%) believe antidepressants effectively relieve symptoms and improve function in chronic pain management. Combining analgesics with different mechanisms for better pain control and fewer side effects garners substantial support (47.9% agree) in statement 2e. Lastly, statement 2f indicates a strong consensus (66.2% agree, 25.4% strongly agree) on the need for individualized opioid analgesic dosing, emphasizing personalized care in response to pain.

### 3.3. Self-Reported Competence in Pain Management 

**Figure 2:**
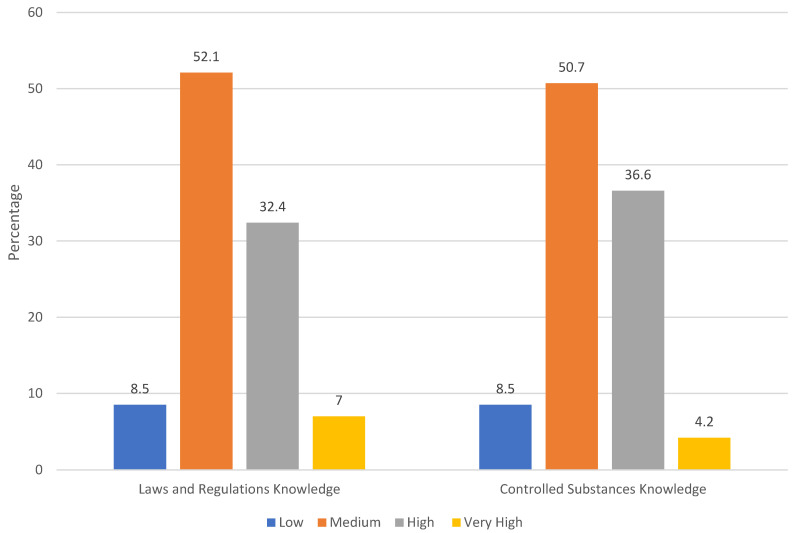
The knowledge of laws and regulations related to controlled substances and pain management.

The knowledge of laws and regulations related to controlled substances and pain management was rated by most respondents as medium to high, while 8.5% rated their knowledge as low (Figure 2).

### 3.4. Self-Reported Competence in Pain Management 

Respondents’ comfort levels were found high in advising on OTC analgesics, confidence in counseling on non-pharmacological strategies, and self-rated knowledge in pain management. The majority felt comfortable advising on OTC analgesics, with 52.1% indicating comfort and 29.6% feeling very comfortable. Similarly, confidence in counseling on non-pharmacological strategies was notable, with 45.1% feeling confident and an additional 11.3% feeling very confident. Moreover, over half of the respondents (54.9%) rated their knowledge of pain management as high, and 5.6% considered it very high.

88.7% of respondents encounter patients seeking advice on non-pharmacological pain management strategies, while 78.9% routinely consider potential interactions with other medications or health conditions when discussing OTC analgesics. Additionally, 59.2% were familiar with guidelines for OTC analgesics in Norway. 50.7% of respondents agree, and 29.6% strongly agree that they could benefit from training in pain management. Furthermore, a majority believe in the utility and importance of clinical guidelines, with 59.2% agreeing they are useful, and 76.1% (66.2% agree, 9.9% strongly agree) emphasizing the importance of adherence to these guidelines. Regarding preference for paracetamol over ibuprofen, 56.3% agree, and 28.2% strongly agree, citing reasons such as paracetamol’s lower risk profile, especially in conditions like pregnancy or gastrointestinal problems, and minimal interactions with other drugs and food. Overall, respondents perceive paracetamol as a safer choice in pain management due to its perceived safety profile and lower risk of adverse effects compared to ibuprofen.

### 3.5. Facilitators and Barriers of Effective Pain Management at Community Pharmacy: Insights from Pharmacists’ Experiences and Perspectives

Notably, 33.8% identified a lack of pain management knowledge, while 23.9% faced communication difficulties, particularly with chronic pain patients. Concerns about opioid dispensing (19.7%) and safety issues (21.1%) were also prevalent, alongside discomfort in advising specific patient groups like pregnant women, children, or the elderly (29.6%). Some respondents (22.5%) reported other challenges, including high costs of pain medications, patient self-management attitudes, and technical prescription issues.

Moreover, 66.2% identified limited time for counseling, high workload (57.7%), and insufficient staffing (62%) as major challenges. Pharmacists also cited issues like outdated training, patient trust, and managing patients across multiple pharmacies. Operational hours, reliance on inexperienced staff, and patient reluctance to discuss pain were notable concerns.

Pharmacy workflow and time constraints significantly impact comprehensive pain management advice, as reported by 25.4% of respondents, while 54.9% indicated some level of impact. It is noteworthy that respondents could select multiple barriers. Table 3 summarizes the findings.

The issue of staffing was further identified with 53.5% indicating that current staffing levels at their pharmacy impact their ability to advise on pain management. 38% of the respondents reported that their pharmacy’s staffing levels are barely sufficient to handle patients’ pain management needs. However, 18.3% reported that the staffing level was not at all sufficient, suggesting a considerable gap in some pharmacies that could affect the quality of pain management advice and service. 

Facilitators for effective pain management are found to have clear guidelines and protocols that received a predominantly positive reception. Almost half (47.9%) of the respondents viewed these guidelines as helpful. Additionally, 22.5% rated them as very helpful, while the same percentage remained neutral.

42.3% of respondents stated that they frequently provide patient education, while another 42.3% mentioned engaging in such activities occasionally. This suggests that most community pharmacists recognize the significance of patient education in pain management and actively contribute to this aspect of patient care.

Responses to the questions on the community pharmacists’ self-perceived effectiveness in assisting patients with pain management revealed that 52.1% of respondents felt they could help patients to a large extent, with an additional 2.8% expressing confidence in their ability to help to a very large extent. Furthermore, 35.2% believed they could assist to neither a small nor a large extent, indicating a moderate level of confidence and belief in their ability to aid patients in pain management.

When asked about factors that facilitate effective pain management in pharmacies, respondents highlighted various key elements. These included the importance of having qualified staff with knowledge of pain management and medications, emphasizing the role of a skilled workforce in providing quality guidance. Personalized approaches tailored to each patient’s specific pain issues and overall health status were also deemed crucial for effective counseling and recommendations. Access to information and training, along with the provision of follow-up services to monitor treatment responses and address concerns, were highlighted as essential. Collaboration with healthcare professionals to ensure consistency in treatment plans was mentioned as significant. Other facilitators included patient compliance, sufficient staffing levels, promotion of topical analgesics, and effective patient counseling. Communication skills, both with customers and within the pharmacy team, were frequently emphasized, along with the importance of continued professional development through training and experience. Some responses suggested the need for better resourcing, such as more time for patient interactions, clearer guidelines, and improved staffing, all aimed at enhancing pharmacists’ role in pain management.

Pharmacists’ perceptions regarding the necessity of additional training in pain management were identified. 59.2% of respondents believed that more training was needed to meet patients’ needs effectively. Even more strikingly, 80.3% expressed a desire to participate in training or courses to enhance their knowledge, skills, and competence in pain management at a pharmacy. This indicates a strong willingness among pharmacists to improve their professional capabilities in this area. Remarkably, no respondents were completely unsure, as there were no percentages reflecting a “Do not know” response, suggesting that pharmacists had a clear stance on their educational needs concerning pain management. A detailed breakdown of the preferred types of training among 64 respondents who elaborated on this matter revealed that online training was the most favored option, with half of the respondents (50%) expressing a preference for this format. Seminars were the next most popular choice, with 32.8% of community pharmacists favoring this form of learning. However, 15.6% of the respondents indicated a preference for thematic evenings as a method of training.

39.4% of the respondents believed that collaboration between community pharmacists and other healthcare professionals in managing chronic pain is effective, while a slightly lower percentage, 33.8%, consider it to be very effective. This indicates a strong consensus on the positive impact of multidisciplinary collaboration on the care of patients with chronic pain.

53.5% of participants reported that they participate less frequently in collaborative discussions on pain management with other healthcare professionals. Additionally, 22.5% of the community pharmacists had never taken part in such discussions. However, 15.5% of the respondents engaged in these collaborative discussions every month.

Table 4 outlines the key facilitators, barriers, and proposed improvement strategies for enhancing pain management practices within community pharmacies.

### 3.6. Association and Impact of Gender, Work Experience, and Education Level on Pharmacists’ Knowledge and Practices in Pain Management

Further analysis was performed and explored how gender, work experience, and education level influence pharmacists’ knowledge and practices in pain management. Results revealed several interesting trends:

Gender did not significantly impact pharmacists’ self-reported knowledge of pain management or their engagement in multidisciplinary collaboration.

Pharmacists with more experience reported lower confidence in advising on non-pharmacological pain management strategies and counseling about the appropriate use of analgesics without prescription. This association was statistically significant.

There was no significant association between education level and pharmacists’ self-rated knowledge of pain management or their understanding of laws and regulations related to controlled substances/narcotics.

Work experience did not significantly influence various other factors like multidisciplinary collaboration, self-rated knowledge of pain management, and perceptions regarding the impact of restricted sales of pain medications.

Table 5 presents the findings. Please note that the Bonferroni correction was applied to adjust *p*-values for statistical significance, leading to the rejection of some null hypotheses. Overall, the findings demonstrated that while certain associations were found, many factors like gender and education level did not have the expected impact on pharmacists’ knowledge and practices in pain management.

We also identified how community pharmacists assess their own understanding of pain management and regulations concerning controlled substances in Norway. Notably, 59.2% of respondents exhibited familiarity with guidelines for OTC analgesics, suggesting a relatively high level of knowledge in this domain. Knowledge of laws and regulations concerning controlled substances and pain management ranged from medium to high. Similarly, respondents’ understanding of controlled substances was predominantly rated as medium to high. Regarding self-assessment, respondents reported an average pain management knowledge score of 3.58, indicating a moderate to high level of self-rated knowledge. Overall, the data suggest that respondents possess a moderately high level of knowledge concerning OTC analgesics, laws and regulations on controlled substances, and pain management.

## 4. Discussion

This study marks the first assessment of community pharmacists’ knowledge, skills, and competence in pain management in Norway, identifying both facilitators and barriers impacting their roles in this regard. By investigating these factors, we aimed to gain insights into pharmacists’ practices and propose enhancements for pain management in community pharmacy settings. In the following, we discuss our findings, relating them to available international research where applicable, despite the lack of local studies.

### 4.1. Demographic Findings

Gender

Our survey reveals a notable predominance of female respondents, consistent with broader trends in the healthcare sector, particularly within pharmacy and nursing [38]. Projections indicate that women will constitute over 70% of the global pharmacy workforce by 2030 [39]. Gender dynamics play a crucial role in various aspects of healthcare, influencing patient care delivery, communication styles, empathy [40], and decision-making processes.

In the context of pain management, females are often perceived as more empathetic, which can significantly benefit patients with chronic pain [41,42]. Female healthcare providers tend to employ more patient-centered communication practices [43,44], potentially leading to enhanced therapeutic outcomes. Our study suggests potential variations in how male and female community pharmacists approach pain management, emphasizing the need for tailored strategies that consider these differences. Addressing gender disparities requires promoting diversity and integrating gender sensitivity modules into pharmacy education and training programs. Further research is essential to fully grasp the impact of gender dynamics on pain management outcomes [45]. Based on our findings we can see equal opportunities [46]. However, future studies could explore other dimensions where gender dynamics might exert a more pronounced influence, informing targeted initiatives aimed at encouraging a diverse range of pharmacists to specialize in pain management and potentially enhance overall patient care.

Age

Our survey indicates a predominantly young workforce, with over half of respondents under 32 years old, suggesting receptiveness to new knowledge and innovations in pain management [47]. Continuous professional development is critical for this demographic to adopt advanced pharmacological treatments and multidisciplinary approaches. Bridging experience gaps through mentorship and practical learning opportunities can enhance patient care outcomes. The impact of age on pain management outcomes warrants further exploration to tailor strategies for workforce development and education [47]. Considering the youthful age and relatively lower confidence levels among freshly graduated pharmacists with limited work experience, there might be a pressing need to enhance their preparedness for dispensing pain medications while prioritizing patient health outcomes. Conducting a behavioral study could provide valuable insights and guide interventions aimed at fostering positive changes in this critical area.

Work experience

A significant portion of our surveyed pharmacists have less than five years of experience, indicating a need for targeted educational efforts in pain management. Encouraging mentorship between experienced and novice pharmacists can bridge this knowledge gap, enhancing patient care outcomes. Our study found that with increasing experience, pharmacists’ confidence in advising on non-pharmacological pain management strategies decreases, highlighting the importance of continuous education that integrates both pharmacological and non-pharmacological approaches.

Implementing ongoing education programs is essential to keep pharmacists updated with the latest pain management practices. Enhancing communication skills, especially in counseling patients about OTC medications, is crucial for improving patient safety. Interestingly, work experience did not significantly affect multidisciplinary collaboration, self-rated knowledge of pain management, or perceptions of the impact of restricted pain medication sales.

In contrast, studies from Saudi Arabia and Nigeria showed that factors like age, nature of work, and work location influence pharmacists’ knowledge and attitudes toward pain management [46,48]. These findings emphasize the importance of continuous education and exposure to diverse healthcare scenarios in enriching pharmacists’ expertise. Early engagement in multidisciplinary teams and policy discussions can be beneficial for developing comprehensive approaches to pain management across all experience levels.

Education level

Our study revealed that the level of education among community pharmacists does not significantly correlate with their self-rated knowledge of pain management or their understanding of laws and regulations concerning controlled substances. However, research from Nigeria highlights the positive impact of higher academic qualifications and clinically related degrees on knowledge scores [46]. To keep pharmacists current, it is essential to integrate specialized courses into pharmacy education and provide continuing education programs.

Emphasizing practical skills such as counseling, patient communication, and ethical considerations in pain management is crucial for preparing pharmacists effectively. Future research could investigate how specialized training and continuing education programs influence pharmacists’ practices in pain management. Pharmacy education institutions play a vital role in ensuring that future pharmacists are well-prepared to address the evolving demands of their profession and provide optimal care to patients.

### 4.2. Competences in Rational Pharmacotherapy and Referral

Integration of survey findings on knowledge, skills, and competence in pain management aligns with current practices emphasizing patient-centered analgesic selection based on pain severity. The World Health Organization (WHO) modified pain ladder recommends a stepwise approach: starting with non-opioids and adjuvants for mild pain, adding weak opioids for moderate pain, and considering stronger opioids alongside interventional therapies for severe pain [14]. This approach supports the survey’s implication that analgesic choice should be guided by pain, promoting cautious opioid use and highlighting non-pharmacological interventions.

Continued education and training, facilitated by platforms like Kompetansebroen and Apokus available in Norway, are crucial. Healthcare professionals need expertise in both pharmacological and non-pharmacological pain management to deliver comprehensive care. This includes facilitating referrals to specialists for patients requiring advanced pain relief strategies. Such a tailored, patient-centric approach enhances pain control, satisfaction, and potentially reduces analgesic misuse [47].

Our survey demonstrated strong support (approximately 80% endorsement) for the early integration of CBT in chronic pain treatment plans. Meta-analyses affirm CBT’s efficacy, particularly for chronic low back pain, in reducing pain perceptions, enhancing quality of life, and preventing psychological conditions like depression and anxiety [33,49]. Early CBT integration [36] that can be delivered remotely and via online platforms (telehealth) [50] may also yield cost savings by reducing long-term pharmacological treatments and healthcare utilization [51]. Empowering patients through CBT promotes engagement, adherence to treatment, and better health outcomes, emphasizing the need for ongoing research in psychological therapies [52]. Advocating for early CBT integration acknowledges chronic pain’s multifaceted nature and supports a holistic, sustainable approach to patient-centered care.

### 4.3. Diverse Perspectives on Pharmacotherapy for Pain Management

Our findings highlight diverse opinions on the efficacy of treating chronic pain solely with analgesics and adjuvant analgesics, with over 40% of respondents endorsing this approach for most patients. This presents the necessity for personalized treatment plans that consider individual needs, conditions, and responses. Recognizing these varied perspectives also demonstrates the importance of tailored assessments to guide the selection of analgesics, antidepressants, and other treatment modalities. Studies emphasize a multimodal approach, integrating both pharmacological and non-pharmacological strategies to comprehensively manage chronic pain [49]. A holistic approach will address physical symptoms and psychological and social dimensions of chronic pain.

Moreover, our findings reveal that more than half of the respondents believe antidepressants generally alleviate symptoms and enhance functionality in chronic pain patients. This viewpoint is supported by the existing literature, particularly for neuropathic pain management [51]. These insights show the complexity of chronic pain management and emphasize the integration of diverse therapeutic strategies. They also highlight the importance of educating pharmacists about various pain management approaches, highlighting the benefits of integrating antidepressants and other non-pharmacological options into treatment plans. This knowledge can, on the other hand, empower patients by informing them about available treatment options and the potential advantages of incorporating antidepressants into their pain management strategies if required.

A recent network meta-analysis highlighted duloxetine as the only antidepressant demonstrating moderate efficacy across all outcomes for chronic pain at standard doses. While promising evidence was found for milnacipran, other antidepressants showed low certainty regarding efficiency in chronic pain management [52].

### 4.4. Perspectives on Guidelines and Model of Care

Guidelines from various international bodies, such as the 2017 Canadian Guideline for Opioids for Chronic Non-Cancer Pain, recommendations from the American Society of Interventional Pain Physicians, and the European Pain Federation, advocate a cautious and evidence-based approach to opioid prescribing [53,54,55,56]. Similarly, the 2022 guidelines from the Centers for Disease Control and Prevention (CDC) emphasize individualizing opioid doses for each patient [57]. Our study aligns with these recommendations, as over 90% of respondents supported adjusting subsequent doses of opioid analgesics based on individual patient responses. Community pharmacists’ knowledge and adherence to guidelines are crucial in implementing this approach effectively and will provide a pathway to enhance patient safety, optimize pain management, and mitigate risks associated with opioid therapy. Personalizing opioid doses allows healthcare professionals to minimize overdose and dependence risks, addressing public health concerns related to opioid misuse. Effective implementation of this approach requires open communication between patients and healthcare providers to adjust dosing based on patient feedback regarding pain relief and side effects and community pharmacists can play a significant role in facilitating this process.

Educating patients about the rationale behind personalized opioid dosing and potential risks empowers them to actively participate in their pain management. Norwegian guidelines on opioid prescription also stress cautious and individualized prescribing, especially for non-cancer chronic pain, advocating limited opioid use and consideration of alternative treatments [58]. These approaches align with international guidelines promoting careful prescribing to mitigate opioid misuse risks. Community pharmacists can play a crucial role in improving outcomes for individuals with chronic pain by raising awareness, advocating for safe and effective pain management practices, and empowering patients. Providing instructions and advice on the proper use, common side effects, and potential drug interactions of long-term analgesics ensures that patients can manage their pain effectively and safely. Consulting with community pharmacists when using long-term analgesics offers patients personalized support tailored to their needs, ultimately enhancing their quality of life despite chronic pain conditions.

### 4.5. Enhancing Community Pharmacists’ Confidence in Non-Pharmacological Pain Management Strategies

Our findings indicate that community pharmacists generally possess a high level of confidence in advising on OTC analgesics, with more than 80% of respondents expressing strong confidence. This proficiency likely stems from pharmacists’ extensive practical experience gained through regular patient interactions in pain management scenarios. Such experiences enable pharmacists to refine their counseling skills, better understand patient needs, and become proficient in managing common pain issues, thus bolstering their confidence in advisory roles.

However, pharmacists with limited experience or infrequent encounters with pain management situations may report lower comfort levels, suggesting a gap in experiential learning. This highlights the potential benefit of additional education or resources to enhance their competence and confidence in providing accurate and beneficial advice.

Moreover, our study demonstrated significant patient interest in non-pharmacological pain management options, with many respondents noting frequent patient inquiries about such strategies. This growing interest emphasizes the importance of pharmacists being well-familiarized or educated in various pain management approaches, including non-pharmacological methods, to effectively guide their patients. Approximately half of the pharmacists expressed moderate to low confidence in offering advice on non-pharmacological pain management strategies. This reflects the necessity for targeted educational interventions. Strategies to improve pharmacists’ proficiency in non-pharmacological pain management can include holding workshops and seminars, where conducting interactive sessions can cover information and awareness about physical therapy, CBT, mindfulness, and dietary approaches. While effective, these events can be time-consuming and costly. Developing online modules focusing on non-pharmacological pain management can also allow pharmacists to learn at their own pace. Online courses are cost-effective and flexible but may lack face-to-face interaction. Facilitating learning sessions with physical therapists, psychologists, and other pain management experts can foster a comprehensive understanding and collaborative network. Challenges include logistical complexities and scheduling conflicts. Utilizing digital platforms to provide accessible information and interactive learning elements like quizzes can be valuable. However, effectiveness can vary based on individual learning styles and the quality of content. Establishing forums where community pharmacists can share experiences and learn from peers in a supportive environment can be vital. This approach is relatively easy to organize and fosters community among pharmacists. A combination of these approaches can provide for diverse learning preferences and needs among community pharmacists. For instance, blending online courses with periodic workshops offers flexibility and practical engagement. Involving community pharmacists in the planning of such activities and co-creation techniques can help ensure that educational interventions are relevant and effective.

Despite challenges such as cost and time constraints, investing in enhancing pharmacists’ confidence and competence in non-pharmacological pain management is crucial. This investment will ultimately enhance patient care within the community pharmacy setting, equipping pharmacists to meet the varying needs of patients effectively and ensuring better pain management outcomes.

### 4.6. Community Pharmacists’ Preferences Versus Evidence on Paracetamol Risks

Our data revealed that a majority of community pharmacists in Norway actively engage in discussions concerning potential interactions between OTC analgesics and patients’ current medications or health conditions, demonstrating a high level of professional commitment to patient safety. However, despite this commitment, over 80% of pharmacists prefer paracetamol over ibuprofen due to its perceived safety profile, fewer side effects, lower gastrointestinal risk, fewer drug interactions, and better tolerance. Contrarily, a systematic review assessing paracetamol’s adverse event profile concluded that higher doses are associated with increased risks of mortality, cardiovascular events, gastrointestinal complications, and renal impairment or failure [59]. This highlights the necessity for continuous education to ensure pharmacists are well-informed about the potential risks associated with higher doses of paracetamol.

Moreover, recent debates about the safety of paracetamol during pregnancy warn about the importance of integrating emerging evidence into educational programs. While commonly believed to be safe, studies suggest potential risks such as increased risks of ADHD in children with prolonged use [60] and possible developmental impacts [61]. Incorporating these findings into educational curricula is crucial for guiding safer medication practices during pregnancy.

Our study also indicated that more than half of the respondents are familiar with guidelines or recommendations related to OTC analgesic use in pain management in Norway, such as the “Guidelines for Pain Relief/Treatment 2009” and the “Norwegian Drug Handbook”. However, nearly half of the respondents were either unaware or unsure about these guidelines, revealing a knowledge gap that requires higher attention. To enhance community pharmacists’ knowledge and skills, organizing events and seminars can be beneficial. Additionally, providing flexible, self-paced online courses and developing comprehensive mobile applications that integrate guidelines, laws, treatment options, interactive learning modules, and community forums can effectively support pharmacists in staying updated and connected. Leveraging AI for evaluating medication safety, drug interactions, and personalized recommendations could further enhance their capabilities.

Furthermore, our findings indicated that Norwegian community pharmacists generally report medium to high knowledge of laws and regulations concerning controlled substances and pain treatment. This suggests that pharmacists are adequately prepared to manage and dispense controlled substances responsibly, which is critical for preventing drug abuse and medication errors. This can be tailored to OTC analgesics. Professional pharmacy organizations play a pivotal role in enhancing community pharmacists’ competencies in pain management by facilitating educational initiatives and providing necessary resources to bridge knowledge gaps, ultimately contributing to improved patient outcomes.

### 4.7. Community Pharmacists’ Self-Assessed Knowledge in Pain Management and Perceived Need for Further Training

Approximately 60% of respondents rated their knowledge as above average, indicating a good to excellent understanding. However, about 40% rated their knowledge as below average to average, revealing clear gaps. Interestingly, 80% of all respondents expressed a need for additional training in pain management, presenting a widespread recognition of this necessity.

To address these gaps effectively, an integrated approach is crucial. This involves regular education for pharmacy students and continuing professional development for practicing pharmacists, facilitated by platforms like Apokus. The curriculum should encompass advanced pharmacology with a focus on pain medications, including opioids, non-opioids, and adjuvant therapies, covering mechanisms of action, pharmacokinetics, and pharmacodynamics. Multimodal pain management must be included with a clear differentiation between chronic and acute pain, managing drug interactions and contraindications, and understanding regulations for treatments. Skills must be mandatory to acquire in patient education, counseling, and communication to engage patients in their pain management plans, adherence, and lifestyle changes, pointing to patient-centric approaches to pain care. It is essential to keep pharmacists updated with the latest guidelines and recommendations through interactive case studies and clinical scenarios. Platforms like Apokus in Norway or European and International platforms for pain education such as the European Pain Federation (EFIC) or the International Association for the Study of Pain (IASP) can deliver structured educational content and certification opportunities for community pharmacists.

Collaboration with pharmacy organizations, such as the International Pharmaceutical Federation (FIP) to offer continuing education credits for pain management courses and promote participation in national and international conferences further enriches learning opportunities. By integrating these strategies into education and professional development plans, community pharmacists can better meet the increasing demand for comprehensive pain management knowledge and skills, while providing services at the community pharmacies.

### 4.8. Community Pharmacists’ Perception of the Importance of Adherence to Clinical Guidelines in Pain Management

Regarding clinical guidelines in pain management, more than 70% of our respondents agreed that they are essential tools for effective treatment. Despite their importance, healthcare practitioners often fall short in adhering to these guidelines [62,63], leading to suboptimal pain management, and potentially causing patient discomfort or adverse effects. Optimal pain management is critical for patient recovery, quality of life, and satisfaction with healthcare services. Clinical guidelines offer evidence-based recommendations on dosages, interactions, and administration routes, influencing medication safety and patient outcomes. Non-adherence increases the risk of medication errors, adverse reactions, and healthcare inefficiencies, including prolonged hospital stays and higher costs.

Moreover, inconsistent adherence contributes to public health challenges like the opioid crisis, exacerbating addiction and overdose rates. Addressing these issues requires continuous education, training, and the implementation of systems that support guideline adherence. Regular audits and patient engagement are essential for monitoring and improving compliance. Professional pharmacy organizations play a crucial role in enhancing pain management practices through education, resource development, and advocacy. They can encourage pharmacists’ involvement in research and promote new findings through educational materials and updated guidelines. Integrating technology such as AI tools and mobile apps can further support pharmacists in delivering evidence-based care and improving patient outcomes. By prioritizing these strategies, pharmacy organizations can most likely empower community pharmacists to provide high-quality, guideline-based care, thereby enhancing patient safety and treatment efficacy in pain management.

### 4.9. Enhancing Pain Management in Community Pharmacies: Barriers, Facilitators, and a Path Forward

Our study identified significant barriers to effective pain management in community pharmacies, including knowledge gaps, communication challenges, concerns about opioid dispensing, staffing limitations, and discomfort in advising vulnerable populations. Addressing these challenges is crucial to improving patient care in chronic pain management. A third of pharmacists lacked adequate knowledge in pain management, impacting patient care and pharmacist confidence. Continuous education and specialized training are essential to address this gap. Nearly a quarter of pharmacists struggled to effectively communicate with chronic pain patients, highlighting the need for enhanced empathy and patient-centered communication skills through workshops and improved collaboration with physicians. The opioid crisis can complicate the perception of community pharmacists to balance between pain relief and misuse prevention. Adherence to guidelines and decision-support tools are vital in navigating these challenges. High workload and limited staffing can affect the quality of pain management advice in community pharmacies. Solutions can include optimizing workflow, hiring additional staff during peak times, and utilizing pharmacy technicians for non-clinical tasks.

Half of our participant pharmacists found guidelines helpful, indicating a desire for evidence-based frameworks in practice. Regular training and involvement in guideline development can enhance their applicability. Pharmacists also play a critical role in patient education. Structured programs and digital resources can standardize and improve educational efforts, in particular in managing pain and analgesic use or referrals. Our community pharmacists also expressed confidence in managing pain but emphasized that they would benefit from ongoing education and mentorship to enhance their skills and efficacy. Clearer guidance and pharmacist involvement in policy discussions are needed for better patient care. Pharmacists in our study self-recognized the need for further training, particularly in emerging therapies and patient counseling. They seemed to prefer online and seminar-based training, highlighting the need for flexible learning options. There was also a consensus on the benefits of interdisciplinary collaboration in pain management, emphasizing the need for formal protocols and regular meetings.

Addressing the identified barriers and enhancing the suggested facilitators in pain management requires collaborative efforts across pharmacy leadership, educational institutions, and healthcare organizations. Collectively, our findings offer actionable recommendations for improving pain management practices in community pharmacies, contributing to broader research on pharmacists’ roles in healthcare. Future studies should consider incorporating patient perspectives and expanding beyond national boundaries for greater applicability. 

### 4.10. Study Limitations and Future Avenues for Further Research

Our study provided insights into community pharmacist perspectives on pain management through a detailed questionnaire, which was constructed after a rigorous process. However, it has several limitations to note, including a potential lack of generalizability beyond Norwegian community pharmacists, absence of direct patient input, modest sample size, and reliance on self-reported data without longitudinal or qualitative methods to delve deeper into responses.

Future studies can benefit from a larger sample size, employing longitudinal research methodologies to track changes in pharmacist practices and knowledge over time, providing insights into trends and the long-term impacts of educational interventions. Qualitative approaches such as interviews or focus groups could offer a deeper exploration of pharmacist experiences and challenges in pain management, complementing quantitative data. International comparative studies would be beneficial to identify common challenges and effective strategies across different healthcare systems. Including patient perspectives in research designs would enhance understanding of patient satisfaction and the effectiveness of pharmacist-led pain management interventions. Additionally, assessing the effectiveness of specific interventions aimed at improving guideline adherence or educational programs among pharmacists could provide valuable insights into optimizing pain management practices. Future studies should also consider involving a broader healthcare community to explore how enhanced collaboration among medical professionals can improve pain management. This research can focus on integrating the capabilities of community pharmacies into a cohesive care model for patients suffering from pain.

## 5. Conclusions

Our study revealed that Norwegian community pharmacists possess a moderate level of knowledge regarding their role in pain management within community pharmacies. However, ongoing education and training programs are found essential to enhance their skills further. Respondents recognized the importance of dispensing pain medication and its vital consequences and emphasized the importance of collaborating with other healthcare professionals to optimize patient care in this context. Additionally, our community pharmacists recognized their pivotal role in ensuring patient safety with over-the-counter analgesics and acknowledged an unmet need to increase awareness of associated risks and updated research-based evidence and new knowledge. They highlighted that continued education and training will empower them to play a more effective role in pain management and improve overall patient care in the community.

## Figures and Tables

**Table 1:** Null hypotheses and statistical tests.

Null Hypothesis	Independent Variable	Type of Independent Variable	Dependent Variable	Type of Dependent Variable	Statistical Test
There is no significant association between gender and self-reported knowledge of pain management.	Gender	Categorical Nominal	Self-reported Knowledge of Pain Management	Categorical Ordinal	Chi-Square Test
There is no significant association between gender/work experience and how often they coordinate with other health care professionals (Multidisciplinary collaboration).	Gender /Work Experience	Categorical Nominal/Continuous	Multidisciplinary Collaboration	Categorical Ordinal	Chi-Square Test/Ordinal Regression
There is no significant association between the work experience of the respondents and their confidence in advising on non-pharmacological pain management strategies.	Work Experience	Continuous	Advising on Non-Pharmacological Pain Management Strategies	Categorical Ordinal	Ordinal Regression
There is no significant association between work experience and counseling about the appropriate use of painkillers without prescription.	Work Experience	Continuous	Counseling about the appropriate use of painkillers without prescription.	Categorical Ordinal	Ordinal Regression
There is no significant association between a pharmacist’s work experience and their self-rated knowledge of pain management.	Work Experience	Continuous	Self-rated Knowledge of Pain Management	Categorical Ordinal	Ordinal Regression
There is no significant association between a pharmacist’s education level and their self-rated knowledge of pain management.	Education Level	Continuous	Self-rated Knowledge of Pain Management	Categorical Ordinal	Ordinal Regression
There is no significant association between pharmacists’ work experience and their views on the impact of restricted sales of pain medication.	Work Experience	Continuous	Views on the impact of restricted sales of pain medication	Categorical Ordinal	Ordinal Regression
There is no significant association between the level of education of pharmacists and their self-rated knowledge of laws and regulations related to controlled substances/narcotics.	Education Level	Continuous	Self-rated knowledge of Laws and Regulations related to Controlled substances/Narcotics	Categorical Nominal	Ordinal Regression

**Table 2:** Socio-demographic characteristics of the respondents.

Information	Total Number of Respondents (n)	Options	Number	Percentage (%)
Gender	71	Male	29	40.8
		Female	42	59.2
		Other	0	0
		Do not wish to answer	0	0
Age (years)	71	21–26	11	15.5
		27–32	27	38
		>32	33	46.5
Work Experience (years)	71	Recent graduate	8	11.3
		<5 years	29	40.8
		5–10 years	13	18.3
		>10 years	21	29.6
Education Level	71	Bachelor’s in pharmacy	43	60.6
		Master’s in pharmacy	25	35.2
		Other	3	4.2
Other Education Level	3	Pharm-D (Doctor of Pharmacy)	2	67
		Master’s in Clinical Pharmacy	1	33
Work Location in Norway	71	Northern Region	9	12.7
		Eastern Region	35	49.3
		Central Region	14	19.7
		Southern Region	7	9.9
		Western Region	6	8.5
Ever received training in Pain Management	71	Yes	45	63.4
		No	17	23.9
		Do not remember	9	12.7
If “Yes”, the source of training received	50	At Pharmacy	23	46
		At University during my studies	22	44
		Other	5	10
If “other”, describe the source of training	5	Both at university and at a pharmacy	1	20
		Other education program	1	20
		Pharmacy training program (Apokus)	1	20
		Had lecture on pain during my studies in my bachelor program, but not direct training	1	20
		Also during studies	1	20
Pharmacy degree completed in Norway	71	Yes	50	70.4
		No	21	29.6
If “NO”, mention the name of the country where the degree in pharmacy was completed	21	Pakistan	15	71
		Egypt	1	4.8
		Ethiopia	1	4.8
		Iran	1	4.8
		Serbia	1	4.8
		Had not mentioned the name of the country	2	9.5

**Table 3:** Assessment of barriers to effective pain management in community pharmacies (n = 71).

Barriers	Number	Percentage (%)
Lack of knowledge about pain management	24	33.8
Communication difficulties	17	23.9
Fear of opioid dispensing	14	19.7
Safety concerns	15	21.1
Uncomfortable during advising to pregnant women, children, or the elderly with chronic pain	21	29.6
Workflow and time availability concerns SIGNIFICANTLY affect the ability to provide comprehensive advice	18	25.4
Workflow and time availability concerns SOMEWHAT affect the ability to provide comprehensive advice	39	54.9
Limited time for counseling	47	66.2
High workload	41	57.7
Lack of staffing	44	62

**Table 4:** The key facilitators, barriers, and proposed improvement strategies for enhancing pain management practices within community pharmacies.

Facilitators	Barriers	Strategies for Improvement
Regular training sessions and workshops on pain management guidelines	Knowledge gaps in pain management	Continuing education and specialized training programs
Structured patient education programs and training in counseling techniques	Communication difficulties with patients suffering from chronic pain	Organizing workshops focusing on empathy, active listening, and patient-centered care
Utilizing digital tools and resources for patient education	Concerns about opioid dispensing and safety issues	Implementing guidelines and employing decision-support tools
Mentorship programs and continuing education opportunities	Limited time, high workload, and inadequate staffing	Workforce planning and pharmacy workflow optimization
Engagement in policy discussions and decision-making	Discomfort advising vulnerable groups on pain management	Targeted training and resources for advising vulnerable groups
Formal channels and protocols for multidisciplinary collaboration	Variance in the perceived helpfulness of guidelines	Ensuring guidelines are accessible, relevant, and tailored

**Table 5:** Results of association analysis of factors influencing community pharmacists’ pain management practices.

Null Hypothesis	Independent Variable	Type of Independent Variable	Dependent Variable	Type of Dependent Variable	Statistical Test	Adjusted *p*-Value (after Bonferroni correction)	Null Hypothesis Rejected/Accepted (With Bonferroni Correction)
There is no significant association between gender and self-reported knowledge of pain management	Gender	Categorical Nominal	Self-reported Knowledge of Pain Management	Categorical Ordinal	Chi-Square Test	0.735	Accepted
There is no significant association between gender/work experience and how often they coordinate with other health care professionals (Multidisciplinary collaboration)	Gender/Work Experience	Categorical Nominal/Continuous	Multidisciplinary Collaboration	Categorical Ordinal	Chi-Square Test/Ordinal Regression	0.171/0.771	Accepted/Accepted
There is no significant association between the work experience of the respondents and their confidence in advising on non-pharmacological pain management strategies.	Work Experience	Continuous	Advising on Non-Pharmacological Pain Management Strategies	Categorical Ordinal	Ordinal Regression	<0.001	Rejected
There is no significant association between work experience and counseling about the appropriate use of painkillers without prescription.	Work Experience	Continuous	Counseling about the appropriate use of painkillers without prescription	Categorical Ordinal	Ordinal Regression	0.003	Rejected
There is no significant association between a pharmacist’s work experience and their self-rated knowledge of pain management.	Work Experience	Continuous	Self-rated Knowledge of Pain Management	Categorical Ordinal	Ordinal Regression	0.021	Accepted
There is no significant association between a pharmacist’s education level and their self-rated knowledge of pain management	Education Level	Continuous	Self-rated Knowledge of Pain Management.	Categorical Ordinal	Ordinal Regression	0.550	Accepted
There is no significant association between pharmacists’ work experience and their views on the impact of restricted sales of pain medication.	Work Experience	Continuous	Views on the impact of restricted sales of pain medication	Categorical Ordinal	Ordinal Regression	0.422	Accepted
The is no significant association between the level of education of pharmacists and their self-rated knowledge of laws and regulations related to controlled substances/narcotics	Education Level	Continuous	Self-rated knowledge of Laws and Regulations related to Controlled Substances/ Narcotics	Categorical Nominal	Ordinal Regression	0.447	Accepted

## Data Availability

Original data are available.

## Supplementary Materials

The following supporting information can be downloaded at: https://www.mdpi.com/article/10.3390/pharmacy12040111/s1, the original Norwegian questionnaire and the English translation.
