# Supplementary material for: Exploring the Role of Community Pharmacists in Pain Management: Enablers and Challenges
Source: Pharmacy (Basel). 2024 Jul 18;12(4):111. doi: 10.3390/pharmacy12040111 (PMC11270416; doi:10.3390/pharmacy12040111)
Supplement: Supplementary file 1 [file pharmacy-12-00111-s001.zip › pharmacy-3097970-supplementary.pdf]

Appendix 3: Questionnaire

Role of community pharmacists in Pain management in Norway

Studien viser at 30% av den voksne befolkningen i Norge lider av kroniske smerter. Mange søker hjelp hos farmasøyter med varierte behov, som inkluderer initiering av smertestillende medisiner, spørsmål angående doseringen og/- eller bivirkninger av analgetika, vurdering av alternative medikamenter, eller å utforske muligheten for å legge til andre medikamenter for å forbedre smertelindringen.
Formålet med vår studie er å kartlegge kunnskapen og holdningene blant apotekfarmasøyter i forhold til smertehåndtering. Spesielt er vi interessert i å evaluere apoteksfarmasøyter kunnskap om smertehåndtering, samt apoteksfarmasøytenes komfortnivå når det gjelder utlevering av smertestillende til pasienter som søker råd eller resept. I tillegg vil vi samle apoteksfarmsøyters meninger og tro på smertehåndtering, samt deres oppfattende hindringer og faktorer som kan bidra til suboptimal funksjon i denne rollen. Gjennom dette ønsker vi å skape større oppmerksomhet rundt dette emnet og øke den praktiske kunnskapen blant apoteksfarmasøyter.

Vi håper derfor at du kan ta deg tid til å gi dine svare. Spørreundersøkelsen vil ta ca 10 minutter å svare på og resultatene fra denne undersøkelsen vil bli brukt i forbindelse med mitt masterprosjekt innen farmasi ved OsloMet, der jeg, Syed Hassan Mujtaba, er masterstudent. Dette prosjektet gjennomføres under veiledning av mitt prosjekt veileder, Parisa Gazerani.
Disclaimer:
Dette er en anonym nettbasert undersøkelse, og SIKT er informert om denne anonyme undersøkelsen. Vi vil ikke samle inn noen av dine personlige eller sensitive opplysninger. For å delta i denne undersøkelsen må du være autorisert farmasøyt og jobber enten i kjedeapotek eller i private apotek. Ved å delta i denne undersøkelsen gir du oss samtykke til å bruke svarene til akademiske formål, forskning og publisering av data i form av en vitenskapelig artikkel.

Er du en autorisert farmasøyt i Norge

Ja

Nei

Jobber du i et kjedeapotek eller i et privat apotek

Ja

Nei

Takk for din interesse for å delta i undersøkelsen. Dessverre samler vi bare data fra apoteksfarmasøyter som jobber i kjede-eller private apotek.

SPØRRESKJEMA:

Spørreskjemaet for studien besto av **4 deler,** som **sosiodemographi, kunnskap, ferdigheter og kompetanse innen smerte og smertebehandling ved hjelp av smertestillende, selvrapportert kompetanse om smertebehandling,** og den siste delen om faktorer som **fremmer eller hindrer apoteksfarmasøyters rolle i smertehåndtering og smertebehandling.**

DEL 1: Sosiodemografiske kjennetegn ved studiedeltakerne:

1. Kjønn:

Mann

Kvinne

Annet

Ønsker ikke å svare

2. Alder

21-26 år

27-32 år

>32 år

3. Arbeidserfaring

Nyutdannet Farmasøyt

<5 år

5-10 år

>10 år

4. Utdanningsnivå

Bachelor i farmasi

Master i farmasi

Annet

4.1 Hvis annet, vennligst beskriv nedenfor

5. Arbeidssted i Norge

Nordlige region

Sentrale region

Østlige region

Vestlige region

Sørlige region

6. Noen gang fått opplæring i smertehåndtering

Ja

Nei

Husker ikke

6.1 Hvis JA, hvor fikk du opplæring

På apoteket

På skole ved studie

Annet

6.1.1 Hvis annet, vennligst beskrive nedenfor

7. Farmasiutdanning oppnådd i Norge

Ja

Nei

7.1 Dersom svaret er Nei, fra hvilket land tok du farmasiutdanningen din

DEL 2: Kunnskap, ferdigheter og kompetanse innen smerte og smertebehandling ved hjelp av smertestillende:

1. "WHO-analgesic ladder" for barn inkluderer ikke svake opioider.

Vennligst velg det alternativet som best beskriver din grad av enighet:

Sterkt uenig

Uenig

Verken enig eller uenig

Enig

Sterkt enig

2. Føler spedbarn mindre smerte enn voksne i lignende situasjoner?

Sterkt uenig

Uenig

Verken enig eller uenig

Enig

Sterkt enig

3. Valg av anbefalt smertestillende bør avhenge av smerteintensitet

Sterkt uenig

Uenig

Verken enig eller uenig

Enig

Sterkt enig

4. Kognitiv atferdsterapi (Cognitive behavioural therapy CBT) er svært effektiv i behandling av kroniske smerter og bør brukes så tidlig som mulig i behandlingsplanen for de fleste pasienter med kroniske smerter

Sterkt uenig

Uenig

Verken enig eller uenig

Enig

Sterkt enig

5. Behandling av kroniske smerter bare med analgetiske og adjuvante smertestillende* er effektivt hos de fleste pasienter

(*Legemidler som opprinnelig ble utviklet for behandling av andre tilstander, men som også har egenskaper som gjør dem nyttige som smertestillende midler for eksempel antidepressiver, kortikosteroider, antiepileptika, gabapentinnoider osv. (https://sml.snl.no/smertestillende_midler)

Sterkt uenig

Uenig

Verken enig eller uenig

Enig

Sterkt enig

6. Bruk av reseptfri Paracetamol er kontraindisert ved astma og lungebetennelse

Sterkt uenig

Uenig

Verken enig eller uenig

Enig

Sterkt enig

7. NSAIDs (Ikke-steroide antiinflammatoriske midler) kan øke risikoen for hjerte- og karsykdommer, inkludert hjerteinfarkt, hjerneslag, hjertesvikt og atrieflimmer

Sterkt uenig

Uenig

Verken enig eller uenig

Enig

Sterkt enig

8. Antidepressiva vanligvis bidrar til å lindre symptomer og forbedre funksjonen hos pasienter med kroniske smerter

Sterkt uenig

Uenig

Verken enig eller uenig

Enig

Sterkt enig

9. Å kombinere analgetika som virker ved ulike mekanismer (f.eks. å kombinere en NSAID med en opioid) kan resultere i bedre smertekontroll med færre bivirkninger enn å bruke en enkelt analgetisk agent.

Sterkt uenig

Uenig

Verken enig eller uenig

Enig

Sterkt enig

10. Etter at en initial dose av en opioidanalgetikum er gitt, bør påfølgende doser justeres i tråd med den enkelte pasients respons

Sterkt uenig

Uenig

Verken enig eller uenig

Enig

Sterkt enig

DEL 3: Selvrapportert Kompetanse om Smertebehandling:

1. Hvor tilfredsstillende synes du at farmasiutdanningen din har vært når det gjelder narkotika/rusmidler og smertebehandling?

Lav

Moderat

Middels

Høy

Svært høy

2. Hvor ofte gir du råd til pasienter om bruk av reseptfrie smertestillende medisiner?

Svært ofte

Ofte

Av og til

Sjelden

Svært sjelden

Aldri

3. Hvor komfortabel føler du deg med å gi råd om riktig bruk av reseptfrie smertestillende medisiner?

(Skala: 1= Ikke komfortable, 5= Svært komfortabel)

4. Har du opplevd at pasienter søker råd om alternative ikke-farmakologiske strategier for smertebehandling, som kalde og varme omslag/ poser, ikke-medisinske geler og medisiner osv

Ja

Nei

Husker ikke

5. Hvor selvsikker føler du deg når du gir veiledning om alternative ikke-farmakologiske smertebehandlingsstrategier?

(Skala: 1= Ikke selvsikker, 5= Svært selvsikker)

6. Når pasienter spør om reseptfrie smertestillende medisiner, diskuterer du ofte mulige interaksjoner med deres andre medisiner eller eksisterende tilstander?

Ja

Nei

Vet ikke

Husker ikke

7. Min generelle preferanse er å anbefale Paracetamol fremfor ibuprofen.

Sterk uenig

Uenig

Verken enig eller uenig

Enig

Sterkt enig

7.1 Hvorfor? Vennligst beskrive nedenfor.

8. Er du kjent med spesifikke retningslinjer eller anbefalinger knyttet til bruk av reseptfrie smertestillende medisiner for smertebehandling i Norge?

Ja

Nei

Vet ikke

9. Din kunnskap om lover og forskrifter knyttet til controlled substances (Narkotika/rusmidler) og smertebehandling.

Lav

Middels

Høy

Svært høy

10. Din kunnskap om controlled substances (Narkotika/Rusmidler).

Lav

Middels

Høy

Svært høy

11. Jeg kunne ha nytte av noe opplæring når det gjelder Smertebehandling.

Sterkt uenig

Uenig

Nøytral

Enig

Sterkt enig

12. Kliniske retningslinjer er NYTTIGE når det gjelder smertebehandling.

Sterkt uenig

Uenig

Verken enig eller uenig

Enig

Sterkt enig

13. Å følge kliniske retningslinjer er VIKTIG når det gjelder Smertebehandling.

Sterkt uenig

Uenig

Verken enig eller uenig

Enig

Sterkt enig

14. Hvordan vil du vurdere din kunnskap om smertebehandling på en skala fra 1 til 5 (1= Lav, 5=Svært høy)

DEL 4: Fremmende og hemmende faktorer for apoteksfarmasøyters rolle i smertehåndtering og smertebehandling:

1. Sammenlignet med andre pasienter, hvilke barrierer/ utfordringer møter du med pasienter som har kroniske smerter? Vennligst merk alle som gjelder

Mangel på kunnskap om smertebehandling

Kommunikasjonsvansker

Frykt for opioidutlevering

Sikkerhetsspørsmål

Ukomfortabel ved rådgivning til gravide, barn eller eldre med kroniske smerter

Ingen av de nevnte utfordringene

Andre

1.1 Hvis annet, vennligst skriv nedenfor

2. Hva er de spesifikke forskjellene du opplever hos pasienter med kroniske smerter sammenlignet med andre pasienter? Vennligst merk alle som gjelder.

Hyppigere bruk av smertestillende medisiner

Mer angst eller depresjon

Behov for spesialisert smertehåndtering

Endring i daglig funksjon eller livskvalitet

Sosiale utfordringer knyttet til smertetilstand

Andre

2.1 Hvis annet, vennligst skriv nedenfor

3. I din mening, trenger apoteksfarmasøyter mer opplæring når det gjelder smerter for å kunne imøtekomme pasientenes behov?

Ja

Nei

Kanskje

Vet ikke

4. Ønsker du muligheten til å delta på en opplæring/ kurs som kan bidra til å forbedre din kunnskap, ferdigheter og kompetanse innen smertehåndtering på et apotek?

Ja

Nei

Kanskje

Vet ikke

4.1 Dersom du svarer ja, kan du fortelle oss hvilken form for opplæring du foretrekker.

Nettbasert

Seminar

Temakveld

Annet

4.1.1 Hvis annet, vennligst spesifiser nedenfor

5. I din mening, hvor effektivt kan en farmasøyt samarbeide med andre helsepersonell for å forbedre omsorgen for pasienter med kroniske smerter?

Svært lite effektivt

Lite effektivt

Verken effektivt eller ineffektivt

Effektivt

Svært effektivt

6. Hvor ofte deltar du i samarbeidende diskusjoner om smertehåndtering med andre helsepersonell?

Daglig

Ukentlig

Månedlig

Sjeldnere

Aldri

7. Hvordan påvirker bekymringer knyttet til apotekets arbeidsflyt og tilgjengelighet av tid din evne til å gi omfattende råd om smertehåndtering?

Påvirker i stor grad

Påvirker noe

Har ingen påvirkning

Vet ikke

8. Hva mener du er den mest betydningsfulle utfordringen knyttet til apoteksfarmasøyter rolle når det gjelder å hjelpe pasienter med smertehåndtering? (Flere alternativer er mulige)

Begrenset tid til rådgivning

Høyt arbeidsvolum

Mangel på bemanning

Ingen av de nevnte utfordringene

Andre utfordringer

8.1 Hvis annet utfordringer, vennligst spesifiser nedenfor

9. Påvirker bemanningen på apoteket din evne til å gi råd om smertehåndtering?

Ja

Nei

Kanskje

Vet ikke

10. Et det tilstrekkelig bemanning i ditt apotek for å håndtere pasienters behov for smertehåndtering?

Ja, mer enn tilstrekkelig

Ja, tilstrekkelig

Nei, knapt tilstrekkelig

Nei, langt fra tilstrekkelig

Usikker

11. I hvilken grad tror du at klare retningslinjer og protokoller for smertehåndtering kan hjelpe i din praksis som farmasøyt?

Svært hjelpsomt
Hjelpsomt
Nøytralt
Ikke så hjelpsomt
Ikke hjelpsomt i det hele tatt

12. I hvilken grad gir du pasientopplæring/ råd for å lette bekymringer og misforståelser om smertestillende medisiner?

Ofte
Av og til
Sjeldnere
Aldri
Usikker

13. I din mening, i hvilken grad kan du hjelpe pasienter med smerter med å forbedre eller lindre smertene deres?

I svært liten grad
I liten grad
Verken i liten eller stor grad
I stor grad
I svært stor grad

14. Begrenset salg av smertestillende medikamenter bidrar til å lette apotekfarmasøyters rolle i smertehåndtering?

Sterkt uenig
Uenig
Verken uenig eller enig
Enig
Sterkt enig

15. Vennligst skriv i boksen nedenfor faktorer som letter effektiv smertehåndtering i et apotek?

Role of community pharmacists in Pain management in Norway

The study shows that 30% of the adult population in Norway suffer from chronic pain. Many seek help from pharmacists with varied needs, which include initiation of pain medication, questions regarding the dosage and/or side effects of analgesics, consideration of alternative medications, or exploring the possibility of adding other medications to improve pain relief. The purpose of our study is to explore the knowledge and attitudes among community pharmacists in regarding pain management. In particular, we are interested in evaluating community pharmacists' knowledge of pain management, as well as the community pharmacists' comfort level when it comes to dispensing painkillers to patients seeking advice or a prescription. In addition, we will gather community pharmacists' opinions and beliefs about pain management, as well as their perceived barriers and factors that may contribute to suboptimal functioning in this role. Through this, we want to create greater attention to this subject and increase practical knowledge among community pharmacists.

We therefore hope that you can take the time to give your answers. The survey will take about 10 minutes to answer and the results from this survey will be used in connection with my master's project in pharmacy at OsloMet, where I, Syed Hassan Mujtaba, is a master's student. This project is carried out under the guidance of my project supervisor, Parisa Gazerani.

Disclaimer:

This is an anonymous online survey, and SIKT has been informed about this anonymous survey. We will not collect any of your personal or sensitive information. To participate in this survey, you must be an authorized pharmacist and work either in chain pharmacies or in private pharmacies.
By participating in this survey, you give us consent to use the answers for academic purposes, research and publication of data in the form of a scientific article.

Are you an authorized pharmacist in Norway?

Yes

No

Do you work in a chain pharmacy or in a private pharmacy?

Yes

No

Thank you for your interest in participating in the survey. Unfortunately, we only collect data from

Community pharmacists who work in chain or private pharmacies.

QUESTIONNAIRE:

The questionnaire for the study consisted of **4 parts,** such as **socio-demography, knowledge, skills and competence in pain and pain management using painkillers, self-reported competence about pain management,** and the last part on factors that **promote or hinder the role of community pharmacists in pain management and pain treatment.**

PART 1: Sociodemographic characteristics of the study participants:

1. Gender:

Man

Woman

Other

Don't want to answer

2. Age

21-26 years

27-32 years

>32 years

3. Work experience

Newly graduated Pharmacist

<5 years

5-10 years

>10 years

4. Level of education

Bachelor in Pharmacy

Masters in Pharmacy

Other

4.1 If otherwise, please describe below

5. Workplace in Norway

Northern region

Central region

Eastern region

Western region

Southern region

6. Ever received training in pain management

Yes

No

Do not remember

6.1 If YES, where did you receive your training?

At the pharmacy

At school when studying

Other

6.1.1 If otherwise, please describe below

7. Pharmacy education obtained in Norway

Yes

No

7.1 If the answer is No, from which country did you receive your pharmacy education

PART 2: Knowledge, skills and competence in pain and pain management using painkillers:

1. The "WHO analgesic ladder" for children does not include weak opioids.

Please select the option that best describes your level of agreement:

Strongly disagree

Disagree

Neither agree nor disagree

Agreed

Strongly agree

2. Do infants feel less pain than adults in similar situations?

Strongly disagree

Disagree

Neither agree nor disagree

Agreed

Strongly agree

3. Choice of recommended pain reliever should depend on pain intensity

Strongly disagree

Disagree

Neither agree nor disagree

Agreed

Strongly agree

4. Cognitive behavioral therapy (CBT) is very effective in the treatment of chronic pain and should be used as early as possible in the treatment plan for most patients with chronic pain

Strongly disagree

Disagree

Neither agree nor disagree

Agreed

Strongly agree

5. Treatment of chronic pain only with analgesic and adjuvant painkillers* is effective in most patients

(*Medicines that were originally developed for the treatment of other conditions, but which also have properties that make them useful as painkillers, for example antidepressants, corticosteroids, antiepileptics, gabapentinoids, etc. (https://sml.snl.no/ smertestillende_midler)

Strongly disagree

Disagree

Neither agree nor disagree

Agreed

Strongly agree

6. Use of over-the-counter Paracetamol is contraindicated in asthma and pneumonia

Strongly disagree

Disagree

Neither agree nor disagree

Agreed

Strongly agree

7. NSAIDs (Non-Steroidal Anti-Inflammatory Drugs) may increase the risk of cardiovascular disease, including heart attack, stroke, heart failure and atrial fibrillation

Strongly disagree

Disagree

Neither agree nor disagree

Agreed

Strongly agree

8. Antidepressants usually help relieve symptoms and improve function in patients with chronic pain

Strongly disagree

Disagree

Neither agree nor disagree

Agree

Strongly agree

9. Combining analgesics that act by different mechanisms (eg, combining an NSAID with an opioid) may result in better pain control with fewer side effects than using a single analgesic agent.

Strongly disagree

Disagree

Neither agree nor disagree

Agree

Strongly agree

10. After an initial dose of an opioid analgesic is given, subsequent doses should be adjusted in line with the individual patient's response

Strongly disagree

Disagree

Neither agree nor disagree

Agree

Strongly agree

PART 3: Self-reported Competence on Pain Management:

1. How satisfactory do you think your pharmacy education has been in terms of narcotics and pain management?

Low

Moderate

Medium

High

Very high

2. How often do you advise patients on the use of over-the-counter pain relievers?

Very often

Often

Occasionally

Rare

Very rare

Never

3. How comfortable do you feel giving advice on the appropriate use of over-the-counter pain medications?

(Scale: 1= Not comfortable, 5= Very comfortable)

4. Have you experienced patients seeking advice on alternative non-pharmacological strategies for pain management, such as cold and hot compresses/pouches, non-medicinal gels and medicines, etc.

Yes

No

Do not remember

5. How confident do you feel when giving advice on alternative non-pharmacological pain management strategies?
(Scale: 1= Not confident, 5= Very confident)

6. When patients ask about over-the-counter pain medications, do you often discuss possible interactions with their other medications or existing conditions?

Yes

No

Do not know

Do not remember

7. My general preference is to recommend Paracetamol over Ibuprofen.

Strongly disagree

Disagree

Neither agree nor disagree

Agreed

Strongly agree

7.1 Why? Please describe below.

8. Are you familiar with specific guidelines or recommendations relating to the use of over-the-counter painkillers for pain management in Norway?

Yes

No

Do not know

9. Your knowledge of laws and regulations relating to controlled substances (narcotics) and pain management.

Low

Medium

High

Very high

10. Your knowledge of controlled substances.

Low

Medium

High

Very high

11. I could benefit from some training in Pain Management.

Strongly disagree

Disagree

Neutral

Agreed

Strongly agree

12. Clinical guidelines are USEFUL when it comes to pain management.

Strongly disagree

Disagree

Neither agree nor disagree

Agreed

Strongly agree

13. Following clinical guidelines is IMPORTANT when it comes to Pain Management.

Strongly disagree

Disagree

Neither agree nor disagree

Agreed

Strongly agree

14. How would you rate your knowledge of pain management on a scale from 1 to 5

(1= Low, 5= Very high)

PART 4: Promoting and inhibiting factors for the role of community pharmacists in pain management and pain treatment:

1. Compared to other patients, what barriers/challenges do you encounter with patients who have chronic pain? Please mark all that apply

Lack of knowledge about pain management

Communication difficulties

Fear of opioid dispensing

Security issues

Uncomfortable when counseling pregnant women, children or the elderly with chronic pain

None of the aforementioned challenges

Others

1.1 If otherwise, please write below

2. What are the specific differences you experience in patients with chronic pain compared to other patients? Please mark all that apply.

More frequent use of painkillers

More anxiety or depression

Need for specialized pain management

Change in daily function or quality of life

Social challenges related to the pain condition

Others

2.1 If otherwise, please write below

3. In your opinion, do communtiy pharmacists need more training regarding pain in order to meet patients' needs?

Yes

No

Maybe

Do not know

4. Do you want the opportunity to participate in a training/course that can help improve your knowledge, skills and competence in pain management in a pharmacy?

Yes

No

Maybe

Do not know

4.1 If you answer yes, you can tell us which form of training you prefer.

Web-based

Seminar

Theme evening

Other

4.1.1 If otherwise, please specify below

5. In your opinion, how effectively can a pharmacist collaborate with other healthcare professionals to improve care for patients with chronic pain?

Very ineffective

Little effective

Neither effective nor ineffective

Effectively

Very effective

6. How often do you participate in collaborative discussions about pain management with other healthcare professionals?

Daily

Weekly

Monthly

Less often

Never

7. How do concerns related to pharmacy workflow and time availability affect your ability to provide comprehensive pain management advice?

It affects to a large extent

It affects to a little extent

Has no influence

Do not know

8. What do you think is the most significant challenge related to the role of community pharmacists when it comes to helping patients with pain management? (Several options are possible)

Limited time for advice

High workload

Lack of staffing

None of the aforementioned challenges

Other challenges

8.1 If other challenges, please specify below

9. Does the staffing at the pharmacy affect your ability to give advice on pain management?

Yes

No

Maybe

Do not know

10. Is there sufficient staffing in your pharmacy to handle patients' needs for pain management?

Yes, more than sufficient

Yes, sufficient

No, barely sufficient

No, far from sufficient

Unsure

11. To what extent do you think that clear guidelines and protocols for pain management can help in your practice as a pharmacist?

Very helpful

Helpful

Neutral

Not so helpful

Not helpful at all

12. To what extent do you provide patient education/advice to ease concerns and misunderstandings about painkillers?

Often

Occasionally

Less often

Never

Unsure

13. In your opinion, to what extent can you help patients with pain to improve or relieve their pain?

To a very small extent

To a small extent

Neither to a small nor to a large extent

Largely

To a very large extent

14. Restricted sales of painkillers help to facilitate the role of community pharmacists in pain management?

Strongly disagree

Disagree

Neither disagree nor agree

Agreed

Strongly agree

15. Please write in the box below factors that facilitate effective pain management in a pharmacy?
